# A conversation with Dr. Judith S. Hochman, Senior Associate Dean of Clinical Sciences, NYU Langone Health

**DOI:** 10.1017/cts.2022.449

**Published:** 2022-08-22

**Authors:** Kathy Siranosian

**Affiliations:** Clinical Research Forum, Washington, DC, USA

## Top 10 Clinical Research Achievement Awards Q & A

This article is part of a series of interviews with recipients of Clinical Research Forum’s Top 10 Clinical Research Achievement Awards. This article is with Dr. Judith S. Hochman, Senior Associate Dean for Clinical Sciences, Co-Director of the NYU-H+H Clinical and Translational Science Institute, Harold Snyder Family Professor and Associate Director of Leon Charney Division of Cardiology, and Director of the Cardiovascular Clinical Research Center at NYU Langone Health. Dr. Hochman, along with Dr. David J. Maron of Stanford University, designed and carried out the ISCHEMIA (International Study of Comparative Health Effectiveness with Medical and Invasive Approaches) trial over more than a decade. The trial examined the impact of adding invasive procedures to guideline-directed medical therapy for patients with stable coronary artery disease and provides important information to use in decision-making between physician and patient on disease management. This study received Clinical Research Forum’s highest honor in 2021, the Herbert Pardes Clinical Research Excellence Award, as the research study that best shows a high degree of innovation and creativity, advances science, and has an impact upon human disease. *The interview has been edited for length and clarity.*

## What were the early influences that shaped your interest in clinical research?

I first became interested in science because of an inspirational biology teacher I had in 10th grade. Back then, I wanted to understand the fundamentals and was very basic science-oriented, which led to my degree in cellular and developmental biology. But I also wanted to work with patients, so I went on to medical school. Eventually, it became clear that the way to combine my interests in science and patient care would be to do clinical research. I was working as director of a cardiac care unit and began participating in clinical trials. That’s when my clinical research career started, and I’ve been passionate about it ever since. There’s a real gap between basic research and when that research translates to improving people’s lives. Clinical trials offer a more direct connection to actually impacting patient care and patient life; they are the gold standard for proving that newly discovered treatments are actually effective and safe. It’s been incredibly rewarding for me to work in this field and be able to integrate my interests. I saw that I could do clinical medicine and oversee the cardiac care unit, while also doing research.

## You mentioned your earlier clinical trial work. How did that impact your work on the ISCHEMIA trial?

I had worked on other clinical trials that were along the same lines as the ISCHEMIA (International Study of Comparative Health Effectiveness with Medical and Invasive Approaches) trial in terms of testing the incremental role of revascularization added to medical therapy for different subsets of patients with ischemic heart disease. Those trials involved patients with cardiogenic shock complication acute MI and stable post MI patients. We designed and executed the ISCHEMIA trial to build on and address limitations of prior trials and to be the definitive trial looking at patients with what was believed to be the highest risk subset: those with extensive ischemia on stress testing.

## How has clinical research changed as you’ve been working on these different trials?

One of the most dramatic changes has been in the size of studies. The ISCHEMIA trial was more than twice the size of the largest prior trial, and it included more than 5000 randomized patients from 37 countries. Many decades ago, we really didn't understand confidence intervals and precision around the estimate of the treatment effect. It’s a different era now. Today’s studies have much larger enrollments with a large number of events targeted, and the vast majority that are impactful are multicenter. That doesn't mean you shouldn't start off with a single center pilot and then expand, but in general, studies are much larger than they were.

Another area that has changed dramatically is what we can learn from many techniques that enable more “deep phenotyping”, including the use of biospecimens collected. Because of the advances in fields like genomics, proteomics, metabolomics, epigenetics, and the sophistication of imaging techniques, we are able to facilitate precision medicine. In the past, we’d get a biospecimen and run some basic lab tests. Now, there is the potential for an array of multi-omic testing. Of course, that means decisions need to be made about prioritization. Funding is not infinite, and when a study is being designed, you have to make choices about what’s potentially the most impactful.

## In light of these changes, what advice do you have for people beginning their careers in clinical research?

Now that clinical trials are so vast in scope, you really have to be a team player. There’s no way you can be a deep expert in every aspect, and you have to be able to collaborate with those who can contribute the expertise you need. The NIH has a website dedicated to the discipline of team science and people who engage in clinical research should really take advantage of those resources [1]. There are so many different parts to a successful collaboration. For example, more than 10 years before we published the main report on the ISCHEMIA trial, my co-chair and I discussed who would be first author on that article. People think that’s wild, but it’s just one example of how you must define the details of who’s doing what. Obviously, there is always potential for change, but you have to set a framework and adhere to that agreement. Large teams need leaders, so people going into this field also should look at building their leadership skills. There’s so much that goes into building teams, managing teams, and driving consensus, and then, ultimately, when there are shades of grey, somebody has to make the decisions. That’s not always an easy position to be in, and if you aspire to a leadership role, you need to learn the techniques required. Granted, not everybody has to be a leader. For many people, being part of the team is rewarding and is sufficiently fulfilling. But if you have leadership aspirations, I recommend taking leadership courses and finding a mentor who’s a leader. Lastly, if you’re going to be an independent clinical investigator, you need to be tenacious and you have to be able to accept a lot of rejection – because rejection is bound to happen. One of the most impactful trials I ran, the Should We Emergently Revascularize Occluded Coronaries for Cardiogenic Shock (SHOCK) trial, had to be submitted three times to get funding from NIH. But I didn't take it personally. I looked through the reviews and said, “Okay, this is a good point. This is a good point. Oh, this one I’ll rebut.” That takes tenacity and resilience.

## How did you learn those skills?

For the tenacity, I think I might have been born with it. But I also had wonderful mentors and role models along the way, and they taught me so much. Eugene Braunwald, MD was Chairman of Medicine when I was a House Officer at the Brigham and Women’s Hospital, and I had the good fortune many years later to be asked by him to serve on a number of committees for trials. That was a wonderful learning experience. After that, Bernadine Healy, MD was my mentor during my cardiology fellowship and she was also instrumental. She certainly was persistent and incredibly accomplished in multiple domains, going on to be the first female director of the NIH and president of the American Red Cross and American Heart Association, among other achievements. My mentors taught me that when you have a passion, you need to pursue it.

## What continues to motivate you?

There’s no doubt that improving the lives of patients is a very strong motivator. In addition, I’m continually motivated by mentoring young people. At NYU, we have a formal mentoring structure for young faculty and there are multiple opportunities for me to informally mentor, as well. The challenges today are extreme. Nobody has enough time and the demands on people’s time are incredible. So, I try to help with prioritization, with efficiency. But there’s also something else. When you're doing a clinical trial, little unexpected things will come up, and there’s nothing like experience to help deal with those. You won't read it in a book. You can't learn it in a classroom. You need experience. That’s why it’s important to have more senior people guiding more junior investigators on clinical trials. Here is one recent example. Early on in the pandemic, there was a huge demand for clinical trials. An infectious disease specialist here was able to pull together a multi-center clinical trial testing convalescent plasma incredibly quickly – because she had help from an experienced team and oversight. From protocol finalization to IRB approval took something like three days, and then the first patient enrolled 2 days later. That was warp speed, and it was only possible because everybody stepped up to contribute their experience and expertise.

## Outside of clinical research, what other activities do you enjoy? How do these activities impact your work?

I like to stay fit, and I work out on the treadmill just about every day. I play tennis when I can. There is good data around how exercise improves cognitive function, so I know this will impact how I age and my work. I also enjoy time with my grandchildren when I have an opportunity to do that, and I enjoy travel. I am very much looking forward to when COVID is in the rearview mirror and I can travel more extensively again.
